# Oxidative Stress in Takotsubo Syndrome—Is It Essential for an Acute Attack? Indirect Evidences Support Multisite Impact Including the Calcium Overload—Energy Failure Hypothesis

**DOI:** 10.3389/fcvm.2021.732708

**Published:** 2021-10-19

**Authors:** Jan Manousek, Petr Kala, Petr Lokaj, Tomas Ondrus, Katerina Helanova, Marie Miklikova, Vojtech Brazdil, Marie Tomandlova, Jiri Parenica, Monika Pavkova Goldbergova, Jiri Hlasensky

**Affiliations:** ^1^Department of Internal Medicine and Cardiology, University Hospital Brno, Brno, Czechia; ^2^Department of Internal Medicine and Cardiology, Faculty of Medicine, Masaryk University, Brno, Czechia; ^3^Department of Biochemistry, Faculty of Medicine, Masaryk University, Brno, Czechia; ^4^Department of Pathophysiology, Faculty of Medicine, Masaryk University, Brno, Czechia

**Keywords:** Takotsubo syndrome, oxidative stress, catecholamines, cytochrome P450, sarco(endo)plasmic reticulum - mitochondria complex, calcium overload, energy failure, antioxidants

## Abstract

Indirect evidences in reviews and case reports on Takotsubo syndrome (TTS) support the fact that the existence of oxidative stress (OS) might be its common feature in the pre-acute stage. The sources of OS are exogenous (environmental factors including pharmacological and toxic influences) and endogenous, the combination of both may be present, and they are being discussed in detail. OS is associated with several pathological conditions representing TTS comorbidities and triggers. The dominant source of OS electrones are mitochondria. Our analysis of drug therapy related to acute TTS shows many interactions, e.g., cytostatics and glucocorticoids with mitochondrial cytochrome P450 and other enzymes important for OS. One of the most frequently discussed mechanisms in TTS is the effect of catecholamines on myocardium. Yet, their metabolic influence is neglected. OS is associated with the oxidation of catecholamines leading to the synthesis of their oxidized forms – aminochromes. Under pathological conditions, this pathway may dominate. There are evidences of interference between OS, catecholamine/aminochrome effects, their metabolism and antioxidant protection. The OS offensive may cause fast depletion of antioxidant protection including the homocystein-methionine system, whose activity decreases with age. The alteration of effector subcellular structures (mitochondria, sarco/endoplasmic reticulum) and subsequent changes in cellular energetics and calcium turnover may also occur and lead to the disruption of cellular function, including neurons and cardiomyocytes. On the organ level (nervous system and heart), neurocardiogenic stunning may occur. The effects of OS correspond to the effect of high doses of catecholamines in the experiment. Intensive OS might represent “*conditio sine qua non”* for this acute clinical condition. TTS might be significantly more complex pathology than currently perceived so far.

## Introduction

### Takotsubo Cardiomyopathy

Takotsubo cardiomyopathy was first described by Sato et al. in 1990 (1). During an acute attack, this heart condition is characterized by transient systolic (and diastolic) dysfunction primarily of the left ventricle, which cannot be explained by coronary angiography. Differential diagnosis must primarily exclude acute myocardial infarction and acute myocarditis (1–3). It is a relatively rare pathology also known by other names, but Takotsubo syndrome (TTS) is recommended as the formal name for this condition (2). Apical ballooning with basal hyperkinesia as a typical regional kinetic abnormality gave the pathology its name (1).

The incidence of TTS is 1–2% of patients with suspected acute coronary syndrome (4, 5). In the USA, this represents 50,000–100,000 cases of TTS per year, and 0.02% of all acute hospitalizations respectively (6, 7). Post-menopausal women are most often affected (89% of all cases), most patients (60%) are older than 65 years (7, 8). Morphologically the most common forms are the apical form with/without involvement of the middle segments of the left ventricle (75–80% of cases) and the midventricular form (10–15%) (2, 7). Several diagnostic criteria have been suggested, including those by the Mayo Clinic (modified in 2008), the Japanese Takotsubo Cardiomyopathy Group, the Gothenburg Group, and the Takotsubo Italian Network (3, 9–12). It is advisable to distinguish two TTS types: primary TTS (heart problems are the primary cause of medical care) and secondary TTS (acute attack occurs in patients originally hospitalized for some other reason) (2).

In a retrospective analysis of 1,109 patients, the most common TTS comorbidities include hypertension (54%), dyslipidemia (32%), mental disorders (24%), smoking (22%), diabetes and obesity (17%), lung disease (15%), malignancies (10%), nervous system and kidney diseases (7%), and thyroid diseases (6%) (13). Smoking, alcohol abuse, hyperlipidemia and anxiety were identified as risk factors for TTS (7).

Acute coronary syndrome has also been described as a possible trigger for TTS. The extent of contractility disorders in these patients with TTS exceeded the supply area from the affected coronary artery (14). However, myocardial infarction due to coronary embolism secondary to TTS has been reported, leading to a cause-and-effect discussion (15, 16).

### Oxidative Stress in Takotsubo Syndrome

Data about oxidative stress (OS) in TTS are scanty. There is a significant disproportion in the number of publications on Takotsubo syndrome/cardiomyopathy and oxidative stress: 5,167 articles about “takotsubo” in the Medline database over the last 20 years, but only 20 articles about “takotsubo oxidative stress” over the past 16 years. References were selected thematically using common web browsers, with no special software used.

Sets of genes controlling the antioxidant response (such as Nrf2-induced genes) have been upregulated in biopsies taken from the myocardium of the small group of patients in the acute phase of TTS, but not after functional recovery. According to the authors, this suggests the importance of OS in the pathophysiology of TTS (17). Other data also point to this possible association (18–21). According to case reports, reviews and systematic reviews, the common characteristics of patients with TTS just before an acute attack seems to be OS, the possible sources of OS have been divided according to its origin into exogenous and endogenous ones (22–24) – Table 1.

**Table 1:** Sources of oxidative stress in acute attack of Takotsubo syndrome.

Exogenous sources (environmental factors)	- Food (e.g., wasabi, shellfish), drinks (e.g., alcohol, energy drinks) - Catecholamines (exogenous source) - Toxins, chemicals, drugs (incl. tobacco, caffeine, cocaine), allergens, vaccines, blood transfusion - Photochemical smog (incl. UV radiation, pollution from transport and industry)
Endogenous sources	- Physical stress - Emotional stress - Catecholamines (endogenous source) - Emergency and acute clinical conditions (incl. infections) - Fenton reaction (i.e., all conditions associated with bleeding including injuries, operations, invasive procedures) - Epileptic seizures - Immunopathological conditions and diseases (incl. acute allergic reactions and delayed-type hypersensitivity) - Acute and chronic inflammations - Chronic disorders (e.g., autoimmune, degenerative, demyelinating, metabolic) - Malignancies

Due to the biochemical pathways, OS is associated with various pathologies or comorbidities potentially leading to TTS. OS sources that can be combined with each other are discussed in detail.

## Oxidative Stress

### Oxidative Stress in Medicine

Oxidative stress as a concept in redox biology and medicine represents “an imbalance between oxidants and antioxidants in favor of the oxidants, leading to a disruption of redox signaling and control and/or molecular damage” (22, 23, 25–27). As the ballast of metabolic processes, OS denotes deviation from redox steady state and evokes stress responses (25).

Under aerobic conditions, more than 90% of the oxygen consumed is reduced directly to water by cytochrome oxidase in the electron transport chain (ETC) using a four-electron mechanism (28–30). ETC located in eukaryotic cells on the inner mitochondrial membrane is associated with oxidative phosphorylation and energy production in the form of ATP from various substrates. Less than 10% of the consumed oxygen is reduced by one-electron conversion to the superoxide radical (O_2_-); by the reduction by another electron and the addition of two protons, hydrogen peroxide (H_2_O_2_) is formed (29–31).

Reactive species (RS) associated with OS can adversely affect biologically important molecules – lipids, proteins and nucleic acids. OS is involved in the development of a number of pathologies such as hypertension, dyslipidemia, metabolic diseases, incl. diabetes mellitus, neurodegenerative disorders, respiratory and inflammatory diseases or malignancies. They also represent TTS comorbidities (7, 13, 24, 32, 33).

In healthy adult women aged 19–78 years, significantly higher levels of lipid peroxidation were found by evaluating the levels of malondialdehyde and F2-isoprostanes. Plasma levels of C-reactive protein (CRP) and cholesterol also positively correlated with both biomarkers (34). Autoantibodies to oxidized DNA were 50 percent higher in women than in men (35). In contrast, ascorbic acid in plasma showed a strong inverse relationship with lipid peroxidation (34). Similarly low concentrations of total glutathione (the most important endogenous antioxidant) were found in the blood of healthy individuals of various ages, primarily those aged between 60 and 79 – see also paragraph 7 (36). These facts suggest a relationship between OS, gender and age, which is important for TTS.

### Oxidative Stress and Reactive Species

RS arise in the body from exogenous and endogenous sources (22–24). They are divided into radicals (so-called free radicals – FR) containing unpaired electrons and non-radicals (22, 37).

The most important RS include reactive oxygen species (ROS) and reactive nitrogen species (RNS) (23, 37). Reactive species of sulfur, selenium, chlorine, bromine or reactive carbonyl compounds such as aldehydes are also known (22). The chemical reactivity of different RS varies by up to 11 orders of magnitude; it is low, for example, for hydrogen peroxide, extremely high for the hydroxyl radical (22). Nitrosative stress served as a modulator of inflammatory changes in the experimental TTS model (19).

More than 90% of ROS is formed in mitochondria, most of which is generated by the escape of electrons from coenzyme Q to molecular oxygen. These electrons are further spontaneously or enzymatically converted to H_2_O_2_ and OH- (29–31). Small amounts of ROS are also produced in the sarco/endoplasmic reticulum, on the plasma and nuclear membranes, and by oxidases (28).

Oxidases oxidizing a number of substrates (carbohydrates, aldehydes, amino acids, heterocyclic compounds, etc.) use electrons to form superoxide radicals (O_2_-). For example, xanthine oxidase (XO) or NADPH oxidase (Nox) are well-known (28). XO metabolizes purines (catalyzes the oxidation of hypoxanthine to xanthine and uric acid) and may be important in patients treated with allopurinol for hyperuricemia or gout. XO-catalyzed conversion of allopurinol (prodrug) to oxypurinol lead to the production of superoxide radicals (38). Under hypoxic conditions, XO itself may be the major source of ROS (39, 40). Nox is responsible, for example, for the inactivation of bacteria and other pathogens by cells of the immune system (39, 40).

Another source of ROS is the autoxidation of small molecules of endo- and exogenous origin such as adrenaline, noradrenaline and many xenobiotics, including drugs (30, 41–44). Other OS sources are also documented (22).

Under normal conditions, there is a dynamic equilibrium between RS production and the antioxidant capacity (45). The ability of cells to respond properly to mild OS is referred to as eustress (46). Short-term OS is important for inducing a process called mitohormesis, where low concentrations of RS act as signaling molecules that initiate the cascade of cellular events and protect cells from harmful effects (47). In general, it has a beneficial effect with ischemic pre- and post-conditioning and prepares the cell for further stress events (48, 49).

When OS disrupts or depletes the antioxidant protection provided by glutathione, antioxidant vitamins, and enzymes (e.g., catalase, peroxidase, superoxide dismutase), protein/enzyme, lipid and DNA tend to be damaged (27). Based on the intensity of the effect, OS can be classified as basal oxidative stress (BOS), low intensity oxidative stress (LOS), intermediate intensity oxidative stress (IOS) and high intensity oxidative stress (HOS); sometimes strong oxidative stress is also described (SOS) (22, 30). Acute, chronic and recurrent OS are distinguished in terms of duration (22).

Oxidative damage products (oxidized forms of lipids, proteins and nucleic acids) can be identified in a laboratory setting (49). Elevated concentrations of 8-hydroxy-2-deoxyguanosine have been found in the urine of patients in acute phase of TTS as an agent of oxidative DNA damage (18). OS also regulates the function of calcium channels and may disrupt the redox state of regulatory proteins in acute TTS attack (21, 50). Damage of cardiomyocytes by OS is manifested by changes in subcellular organelles with intracellular excess of Ca^2+^ (18).

In myocardial biopsies taken in the acute phase of TTS, the whole sets of genes have been triggered by oxidative stress, e.g., genes induced by the transcription factor Nrf2 controlling the antioxidant response by induction of superoxide dismutase (SOD), catalase (CAT) and glutathione peroxidase (GPX) (17, 50). At the same time, protein biosynthesis was significantly overrepresented, transcription of GPX1 and CAT increased (17).

Finally, the effect of OS is manifested by impaired myocardial metabolic function with potassium depletion, depletion of macroergic phosphates, increased intracellular calcium concentration, increased myocardial diastolic tension, and reduced heart contractility (51, 52).

## Exogenous Sources of Oxidative Stress in Takotsubo Syndrome

Exogenous sources penetrate the body from the external environment, where they are metabolized or lead to another endogenous response – Table 1.

### Drugs

A link between drug effects and cardiac pathologies including TTS was mentioned (53). Many molecules interfere with one or more complexes in the respiratory chain, the mitochondrial toxicity of other drugs depends on the production of FR, increased OS, and reduced anti-oxidative capacity (53, 54). The toxic effect of other drugs consists in the impairment of the oxidative phosphorylation, the Krebs cycle, β-oxidation of fatty acids, or in the induction of apoptosis (53).

Drugs are listed as possible triggers of acute attacks of TTS (so-called drug induced TTS) and may represent important indirect evidence of the relationship to OS. Fifty eight cases of TTS have been described in association with twenty different drugs (55). In most cases (77.6%) iatrogenic TTS attack was associated with direct or indirect-acting sympathomimetics (55). Other drugs are presented in this context (55–58). The attention has also been focused on chemotherapy-induced TTS (59, 60). The individual drugs associated with TTS are listed in Table 2.

**Table 2:** Drugs associated with an acute attack of Takotsubo syndrome and their interactions with cytochrome P450 (available from: https://www.drugbank.ca/drugs, https://drug-interactions.medicine.iu.edu/MainTable.aspx).

**Drug**	**Drug characteristic**	**Chemical formula**	**Substrate for cytochrome P450 isoform or other**	**Other CYP/enzyme interaction**	**CYP inhibitory promiscuity**	**Half life**
5-fluorouracil	Pyrimidine analog	C_4_H_3_FN_2_O_2_	P450 1A2, 2A6, 2C8	P450 2C9 (↓)	Low	10–20 min
Albuterol	β2-adrenergic receptor agonist	C_13_H_21_NO_3_		P450 3A4 (↓)	Low	2.7–5 h
Allopurinol	Hypouremic agent	C_5_H_4_N_4_O		Aldehyde oxidase, XO	Low	1–2 h
Anagrelide	Thrombocytosis/chronic myeloid leukemia treatment	C_10_H_7_Cl_2_N_3_O	P450 1A2, 3A4	P450 1A2 (↓)	Low	1.3 h
Atorvastatin	Statin, lipid-lowering drug	C_33_H_35_FN_2_O_5_	P450 3A4, 3A5, 3A457, 3A7, 2C8	P450 2C8 (↓), 2D6 (↓), 2C9 (↓), 2C19 (↓), 2B6 (↑)	High	14 h
Atropine	Cholinesterase inhibitor	C_17_H_23_NO_3_	Muscarinic acetylcholine M1-5 receptors	Not applicable	Not applicable	20 min
Axitinib	Tyrosine kinase inhibitor	C_22_H_18_N_4_OS	P450 3A4, 3A5, 1A2, 2C19		Not applicable	2.5–6.1 h
Bevacizumab	Monoclonal antibody	C_6538_H_10034_N_1716_O_2033_S_44_		Complement, immunoglobulins		
Bleomycin	Antibiotic, antineoplastic	C_55_H_84_N_17_O_21_S_3_	P450 3A4		Low	115 min
Capecitabine	Chemotherapeutic	C_15_H_22_FN_3_O_6_	P450 2B9	P450 2C9 (↓↓)	Low	45–60 min
Carboplatin	Organoplatinum, antineoplastic agent	C_6_H_12_N_2_O_4_Pt	Myeloperoxidase; Glutathione S-transferase theta-1, P, Mu 1; Metallothionein-1A, 2; NAD(P)H dehydrogenase	XO (↑); SOD Cu-Zn (↓)	Low	1.1–2 h
Cefotiam	Cephalosporin	C_18_H_23_N_9_O_4_S_3_	P450 3A4		Low	1 h
Cetuximab	Monoclonal antibody	C_6484_H_10042_N_1732_O_2023_S_36_	Not applicable	Complement, immunoglobulins	Not applicable	75–188 h
Cisplatin	Organoplatinum, antineoplastic agent	Cl_2_H_6_N_2_Pt	Glutathione S-transferase theta-1, P, Mu 1; Metallothionein-1A, 2; Superoxide dismutase Cu-Zn; NADPH dehydrogenase	Myeloperoxidase (↑), XO (↑), P450 4A11 (↑), 2C9 (↓), 2B6 (↓), cholinesterase (↓), Prostaglandin G/H synthase 2 (↓)	Low	30 min
Combretastatin	Antimitotic agent	C_18_H_22_O_6_	Cytochrome c release		Not applicable	Not applicable
Cortisol	Steroid hormone	C_21_H_30_O_5_	P450 3A4, 3A5, 3A7, 11B1, 11B2, Corticosteroid 11-beta-dehydrogenase	P450 3A4 (↑), 2C8 (↑), 2A6 (↑), 1B1 (↑), 2B6 (↑), 2C9 (↑), 2C19 (↑)	Low	2.15 h
Cyclophosphamide	Chemotherapeutic, immunosuppressive agent	C_7_H_15_Cl_2_N_2_O_2_P	P450 2B6, 2C9, 2C19, 2A6, 2C18, 3A5	P450 3A4 (↑), 2B6 (↑), 2C8 (↑)	Low	3–12 h
Cytarabine	Chemotherapeutic, Pyrimidine nucleoside analog	C_9_H_13_N_3_O_5_	P 450 3A4		Low	10 min
Daunorubicin	Chemotherapeutic, anthracycline aminoglycoside	C_27_H_29_NO_10_	P450 3A4, 1A1, 1B1; Xantine oxidase; NADPH-cytochrome P450 reductase	P450 3A4 (↓), 3A5 (↑)	Low	18.5 h
Dipyridamole	Phosphodiesterase inhibitor	C_24_H_40_N_8_O_4_		P450 2C9 (↓), 2D6 (↓), 3A4 (↓)	Not applicable	40 min
Dobutamine	Synthetic catecholamine	C_18_H_23_NO_3_	P450 2D6	COMT	Low	2 min
Docetaxel	Anti-mitotic chemotherapy	C_43_H_53_NO_14_	P450 3A4, 3A5, 3A7, 1B1	P450 3A4 (↓)	Not applicable	4 min−11.1 h
Duloxetine	Serotonin/norepinephrine reuptake inhibitor	C_18_H_19_NOS	P450 2D6, 2C9, 1A2	P450 2D6 (↓), 3A4 (↓), 1A2 (↓), 2B6 (↓), 2C19 (↓)	Not applicable	8–17 h
Epinephrine	Catecholamine, hormone	C_9_H_13_NO_3_		P450 2C9 (↓), 3A4 (↓); COMT, MAO	Low	2–3 min
Etoposide	DNA synthesis inhibitor	C_29_H_32_O_13_	P450 3A4, 1A2, 2E1, 3A5; Glutathione S-transferase theta-1, P	P450 3A4 (↓,↑)	High	4–11 h
Flecainide	Anti-arrhythmic	C_17_H_20_F_6_N_2_O_3_	P450 2D6	P450 2C9	Low	12–27 h
Interferon alpha-2b	Human leukocyte protein moiety	C_860_H_1353_N_229_O_255_S_9_		P450 1A2 (↓)	Not applicable	2–3 h
Interleukin-2	Cytokine, antineoplastic agent	Not applicable	Not applicable	Not applicable	Not applicable	Not applicable
Irinotecan	Antineoplastic enzyme inhibitor	C_33_H_38_N_4_O_6_	P450 3A4, 3A5, 3A7, 2B6	Glucuronyltransferase 1-1, 1-9, Cocaine-esterase	High	6–12 (10–20) h
Ibrutinib	Tyrosine kinase inhibitor	C_25_H_24_N_6_O_2_	P450 3A4, 3A5, 2D6		Not applicable	4–6 h
Ipilimumab	Monoclonal antibody	C_6572_H_10126_N_1734_O_2080_S_40_		Cytotoxic T-lymphocyte protein 4		14.7 days
Levothyroxine	Synthetic thyroid hormone	C_15_H_11_I_4_NO_4_		P450 2C8 (↓)	Low	6–7 days
Lomustine	Alkylating agent, chemotherapeutic	C_9_H_16_ClN_3_O_2_		P450 3A4 (↓), 2D6 (↓)	Low	94 min (16–48 h)
Lumiracoxib	NSAID	C_15_H_13_ClFNO_2_	P450 2C19	P450 1A2 (↓), 2C9 (↓)	Low	4 h
Methotrexate	Cytostatic, antirheumatic agent	C_20_H_22_N_8_O_5_	P450 3A4	e.g. Dihydrofolate reductase, Aldehyde oxidase, Methylenetetrahydrofolate reductase	Low	3–10 (8–15) h
Metoprolol	Betablocker	C_15_H_25_NO_3_	P450 2D6, 3A4	P450 2D6 (↓)	Low	3–7 h
Mitomycin	Cytostatic	C_15_H_18_N_4_O_5_	NADPH-cytochrome P450 reductase, P450 3A4		High	8–48 min
Nivolumab	Monoclonal antibody	C_6362_H_9862_N_1712_O_1995_S_42_		Programmed cell death protein 1		20 days
Norepinephrine	Catecholamine	C_8_H_11_NO_3_		COMT, MAO	Not applicable	Not applicable
Nortriptyline	Tricyclic antidepressant	C_19_H_21_N	P450 2D6, 1A2, 2C19, 3A4, 3A5, 2E1	P450 2D6 (↓)	Not applicable	16–38 h
Oxaliplatin	Organoplatinum, antineoplastic agent	C_8_H_14_N_2_O_4_Pt	Glutathione S-transferase theta-1, P, Mu 1; Metallothionein-1A, 2; Superoxide dismutase Cu-Zn, Myeloperoxidase, NADPH dehydrogenase		Low	0.43/16.8 h/391 days
Oxycodone	Analgesic	C_18_H_21_NO_4_	P450 2D6, 3A4, 3A5		Low	3.2–4.5 h
Pazopanib	Cytostatic	C_21_H_23_N_7_O_2_S	P450 3A4, 1A2, 2C8	P450 3A4 (↓), 2D6 (↓), 2C8 (↓)	Low	35 h
Pembrolizumab	Monoclonal antibody	C_6504_H_10004_N_1716_O_2036_S_46_		Programmed cell death protein 1	Not applicable	26 days
Prednisone	Steroid hormone	C_21_H_26_O_5_	P450 3A4	P450 2A6 (↑), 3A4 (↑), 3A5 (↑), 1B1 (↑), 2B6 (↑), 2C8 (↑), 2C9 (↑), 2C19 (↑), Corticosteroid-11beta-dehydrogenase	Low	2–3 h
Rituximab	Monoclonal antibody	C_6416_H_9874_N_1688_O_1987_S_44_		B-lymphocyte antigen CD20	Not applicable	18–32 days
Sunitinib	Tyrosine kinase inhibitor	C_22_H_27_FN_4_O_2_	P450 3A4, 3A5, 3A7	P450 3A4 (↓)	High	40–60/80–110 h
Trastuzumab	Monoclonal antibody	C_6470_H_10012_N_1726_O_2013_S_42_		Monoclonal antibody against the HER-2 receptor transmembrane tyrosine kinase receptor	Not applicable	28 days
Venlafaxine	SNRI antidepressant drug	C_17_H_27_NO_2_	P450 2D6, 3A4, 2C19, 2C9		Low	5 h
Metformin*	Antidiabetic agent, biguanide	C_4_H_11_N_5_	No. (Excreted as unchanged drug in the urine).	5′-AMP-activated protein kinase subunit beta-1 (↑), Electron transfer flavoprotein-ubiquinone oxidoreductase (↓), Glycerol-3-phosphate-dehydrogenase (NAD+) (↓)		6.2 h

(↓) – enzyme inhibitor; (↑) – enzyme inductor; (*) – see section Chronic Diseases.

Comorbidities of TTS are treated with a number of drugs that are not commonly mentioned in the acute phase (55, 59, 60). From the point of view of OS and drug metabolism, there is a significant association with cytochrome P450 function, there are many interactions between cytochrome P450 and drugs as substrates and/or inducers: e.g., 5-fluorouracil, atorvastatin, axitinib, cortisol, cyclophosphamide, daunorubicin, docetaxel, etoposide, irinotecan, ibrutinib, nortriptyline, oxycodone, pazopanib, prednisone, sunitinib, and venlafaxine. Some interactions with cytochromes are dominated by cyclophosphamide and the glucocorticoids cortisol and prednisone (Table 2). Some TTS patients with malignancies have been treated with the combination of several anticancer drugs which potentiates the interference with cytochromes (59, 60). This is not a typical manifestation of cardiotoxicity, because the clinical outcome is the TTS phenotype (61, 62). Glucocorticoids, if administered briefly and in low doses, lead to a slight increase in OS; with long-term administration, the burden of OS increases dramatically (63). Cortisol-related TTS attack has been reported (64).

Monoclonal antibodies and organic platinum derivatives that do not interfere with CYP's are administered as the part of anti-cancer treatment protocols. They can increase the burden of OS by other mechanisms. Their contribution to the development of TTS cannot be ruled out.

Monoclonal antibodies are degraded to protein fragments and gradually eliminated from the body without the involvement of cytochromes (Table 2). Their molecules containing thousands of oxygen and nitrogen atoms are associated with OS by various mechanisms (65).

Organic platinum derivatives represent substrates for enzymes accelerating OS (XO, myeloperoxidase), but also antioxidants (glutathione S-transferase, metallothioneins and superoxide dismutase Cu-Zn). GST has an important role in the protection against OS – it detoxifies a number of electrophilic compounds (e.g., as oxidized lipids, DNA, catechol products) (66, 67). Some alleles for GST (e.g., GSTM1, GSTT1) encoding lower catalytic activity of the enzyme are associated with increased susceptibility to the toxic effects of xenobiotics, but also with malignancies and asthma (66–68). Generally, SOD Cu-Zn (SOD1) is a soluble cytoplasmic and mitochondrial intermembrane protein, acting to convert superoxide radicals to molecular oxygen and hydrogen peroxide. Hydrogen peroxide can then be broken down by another enzyme - catalase (CAT) (69).

### Cytochrome P450 and Polymorphisms

The human enzyme metabolizing 50–60% of known drugs is cytochrome P450 (CYP) 3A4 (70–72). It belongs to the group of heme thiolate monooxygenases located in the mitochodria or in the liver microsomes / sarco/endoplasmic reticulum. It is involved in NADPH-dependent electron transport, where it ensures the reduction of molecular oxygen to ROS and water (70, 73). CYP 3A4 catalyzes the oxidation of a number of structurally unrelated compounds, including steroids, fatty acids and xenobiotics (70, 74). As an epoxygenase, it also interferes with the metabolism of arachidonic acid, the derivatives of which are associated with the occurrence of malignancies (74).

In relation to OS, substrates and inducers of cytochromes could be important, which increase the metabolic load and thus the production of RS. Well-known inducers of CYP 3A4 are lipophilic substances (phenobarbital, phenytoin), cigarette smoke and ethanol (risk factors for TTS), glucocorticoids, and a number of drugs (70). In contrast, macrolide antibiotics, anti-HIV drugs, antidepressants, calcium channel blockers, steroid modulators, glitazones, etc. represent clinically important inhibitors of the CYP 3A4 isoenzyme (70–72). Cytochrome P450 shows a polymorphism depending on the race, sex, age and tissue in which it is found (72, 75, 76). It is more often expressed in older age, isoforms CYP 2A6 and 2B6 are more common in women (75).

It is known that CYP 2J2 isoform is most expressed in heart tissue (72, 77, 78). This isoenzyme is also associated with the epoxidation of arachidonic acid to signaling molecules and involved in the biotransformation of xenobiotics (77). Its regulation is ensured by stress stimuli, including pro- and antioxidant effects and pro-inflammatory cytokines (79). Left ventricular tissue samples in women showed higher mRNA expression for CYP 2J2 than in men (77). The isoform is associated with hypertension, type 2 diabetes, respiratory pathologies, and the metabolism of xenobiotics, which are the comorbidities of TTS (77). In contrast, the testing of CYP's with various substrates showed low activity of CYP 3A isoforms in the heart (78).

### Catecholamines

The effect of catecholamines on the cardiomyocytes is mediated by the activation of β-adrenergic receptor G protein-coupled receptor kinase 2 (βAR-GRK2). The interaction induces the activation of the coupled G protein into the stimulatory form (Gs protein form) (80). This is followed by the cascade of reactions with the activation of so-called “second messengers,” including enzymes incorporated into the plasma membrane (adenyl cyclase, phospholipase c). The effect of adenyl cyclase is an increase in the level of intracellular cyclic AMP, which activates protein kinase A (PKA) with the subsequent phosphorylation of several intracellular targets, leading to an increased contractile response (80).

#### Catecholamines in Takotsubo Syndrome

One of the most discussed mechanisms of TTS is the myocardial toxicity of catecholamines. In 2008, it was hypothesized that the high levels of circulating adrenaline led by βadrenergic receptor (βAR) activation to the shift in coupled intracellular signal transmission from the stimulatory form of Gs protein to the inhibitory Gi form (81). The result of this change is a negative inotropy with maximal effect in the apical myocardium causing an apical form of the disease, which was confirmed in an animal model for the high bolus dose of adrenaline (81, 82). Differences in regional left ventricular contractility in different forms of TTS were explained by the existence of an apical-basal gradient in βAR density in the left ventricle (the density of βAR in the myocardium decreases from the apex to the heart base) (81). Reports about recurrences of different forms of TTS in the same patients suggest rather a homogeneous distribution of βAR in the myocardium (83, 84).

In the experiment on age-related rat ventricular myocytes, the attenuation of the contractile response to βARs stimulation was demonstrated. This suggests that the positive inotropic effects of βAR stimulation decrease significantly with the age in rat ventricular myocytes, accompanied by a decrease in receptor density and a decrease in membrane adenyl cyclase activity (85).

Also in pathological conditions, the density of βAR in the myocardium changes. Following acute myocardial infarction without heart failure, βAR density is reduced and this reduction predicts later left ventricular dilatation (86). Decreased expression of the βAR-GRK2 complex in lymphocytes has been experimentally demonstrated in inflammatory processes (e.g., arthritis). OS in lymfocytes induced by the exposure to H_2_O_2_ resulted in a 50% reduction in GRK2 protein levels and its activity. The authors consider OS to be a new mechanism for regulating the intracellular activity of GRK2 in inflammation (87).

A review article from 2018 showed that TTS patients have a normal or only a mild to moderate increase in plasma adrenaline at the time of an acute attack (88). Similarly, mild to moderate elevated plasma concentrations of noradrenaline and normetanephrine were found in TTS patients (89). Some TTS patients with heart failure also showed only a mild to moderate elevation of plasma noradrenaline (89). Catecholamines appear to be rather triggers than the cause of an acute attack of TTS (88). There are probably other factors that determine the development of an acute attack of TTS.

In addition to cardiac function and hemodynamics, catecholamines also affect metabolic pathways (90). These effects take place in the context of the “*flight and fight”* response. In general, catecholamines stimulate aerobic glycolysis, glycogenolysis, gluconeogenesis, and inhibit glycogen synthesis (90). Besides, catecholamines enhance ketogenesis and are involved in proteolysis in order to provide sufficient glucose precursors. Their impact on mitochondria is pronounced during critical illness. Here, catecholamines promote mitochondrial uncoupling, and aggravate OS, either *via* an accelerated glycolytic pathway, or through the catecholamine oxidation. This may result in exhausted antioxidant defense systems, such as reduced glutathione (GSH) or SOD, and the progression of mitochondrial dysfunction (90). The metabolic effects of catecholamines in TTS may still be neglected.

In terms of cardiac metabolism, catecholamines act by two rnechanisms. One is by the augmenting the supply of oxidizable substrate. Secondly, the utilization of this substrate by cardiac muscle is influenced by the interaction between oxygen and substrate supply and the contractile state of the heart (91). Catecholamine stimulation is accompanied by the increased use of exogenous energy substrates, especially free fatty acids (FFA). These lipolysis products and beta-oxidation substrates are preferentially used in cardiac metabolism (91). In well-oxygenated cardiac tissue with high concentration of ATP, catecholamines do not lead to the utilization of glycogen (91). In relation to OS, in isolated rat cardiac mitochondria operating in the forward electron transport mode, unsaturated fatty acids increase ROS production by partially inhibiting electron transport and possibly altering membrane fluidity (92).

#### Catecholamines and Oxidative Stress

The biodegradation of catecholamines in the body is ensured by the enzymes COMT and MAO (52, 93). Under normal conditions, 95% of adrenaline and noradrenaline are biotransformed and excreted in the urine in the form of final metabolites (94).

Under pathological conditions (e.g., inflammation), biotransformation of catecholamines *via* oxidation may predominate (93–97). The resulting products are aminochromes (93, 96). They are formed from their catecholamine precursors by several oxidation pathways, which can be an important source of OS (30, 41, 42, 96). The best known products are adrenochrome and its derivative adrenolutin, that can be detected spectroscopically, chromatographically and by nuclear magnetic resonance spectroscopy (93, 96). Aminochromes in TTS have not yet been investigated.

In the experiments, the hypoxanthine-XO system forming ROS can be used to autooxidize adrenaline (97, 98). Yet, in the presence of uric acid (end product of hypoxanthine oxidation) with/without XO, no reaction occurs; this indicates the importance of ROS (98). In a similar experiment on isolated rat cardiomyocytes, there was a concomitant decrease in GSH (99). A membrane system with NADH/NADPH on separated bovine cardiac sarcolemma also induces adrenochrome formation (100, 101). In this system, Nox contributes to the production of adrenochrome by the formation of ROS and NADPH dramatically stimulates adrenaline oxidation (100). The reaction is strongly suppressed by antioxidants such as SOD, ascorbic acid, α-tocopherol or preincubation of the membrane with the beta-blocker propranolol (101).

Cells of the immune system (e.g., polymorphonuclear cells, monocytes, macrophages, eosinophils) also form RS and oxidize adrenaline to adrenochrome (95). After activation of polymorphonuclears, its metabolism proceeds mainly (more than 80%) *via* adrenochrome formation (see also section Allergic Reactions). This pathway is inhibited by SOD, CAT or sodium azide (peroxidase and cytochrome oxidase inhibitor) (95). This may be important in infections and inflammation as comorbidities of TTS (13, 102).

Dobutamin as a synthetic catecholamine can also be oxidized to adrenochrome. “*In vitro*,” this oxidation reaction in the presence of H_2_O_2_ is catalyzed by the enzyme horseradish peroxidase, where more than 95% of the substrate is processed in 5 minutes (103). The enzyme is contained, for example, in wasabi, the consumption of which immediately caused an acute TTS (104, 105). In practice, a substrate for the enzymatic oxidation by horseradish peroxidase could be a native catecholamine. Adrenochrome is also likely to occur during organophosphate poisoning, which has been identified as one of the TTS triggers (95, 106).

Aminochromes are cardiotoxic, neurotoxic and psychotomimetic (52, 95, 96, 103, 107). In experiments, adrenochrome acted on various cellular organelles (108). Effects on the sarco/endoplasmic reticulum and mitochondrial Ca^2+^ transport systems were similar to those of high catecholamine concentrations (108–110). They may explain intracellular and mitochondrial calcium overload with alterations in oxidative phosphorylation, decreased myocardial ATP/AMP ratio, and worsened myocardial contractility (109, 111). Adrenolutin also shows negative inotropy (95). In rat hearts perfused with adrenochrome, signs of necrotic damage such as the dissolution of contractile fibers, disruption and mitochondrial edema, endo/sarcoplasmic reticulum edema were visible by means of electron microscopy (112). These findings are similar to those found in cardiac biopsies of TTS patients (113–115).

In the body, aminochromes are efficiently conjugated to glutathione. The reaction is catalyzed by GST, which prevents redox cycling and shows one of its cytoprotective effects (107). These facts associate the metabolism of catecholamines with OS, and antioxidant protection, respectively.

### Food, Drinks, Drugs, Toxins, and Chemicals

A high-fat and energy-dense diet intensifies the OS (49, 116). Glycemic load and glycemic index are associated with lipid peroxidation (49, 117). While saturated fatty acids potentiate OS, monounsaturated fatty acids, antioxidant vitamins and bioactive substances from fruits and vegetables have antioxidant effects (49). Some TTS attacks occurred during parties and are explained by positive emotions as “*happy heart syndrome”* (118). In these situations, increased energy intake can be expected, which may be related to specific forms of OS - nutritional OS (NOS) or post-prandial OS (POS) (30). Dietary OS (DOS) has also been described (30).

An acute TTS attack after consuming Japanese horseradish - wasabi (*Wasabia japonica, Cochlearia wasabi*) at a wedding party was described (105). The biologically active substances of wasabi are allyl and other isothiocyanates, which generally have antioxidant, neuroprotective and anticancer effects (119–121). Isothiocyanates are rapidly conjugated to glutathione in the liver, metabolized by the mercapturic acid pathway and excreted in the urine; the enzyme gamma-glutamyltransferase, glutathione and xenobiotic load play an important role (119). This can reduce the pool of free glutathione, which is the major endogenous antioxidant (122). Isothiocyanates are eliminated in parallel. A possible end effect may be an alteration in antioxidant protection. Mercapturic acid is also involved in the metabolism of xenobiotics to form reactive thiols (123). This may be another mechanism of action of wasabi (see also section Catecholamines and Oxidative Stress).

Consumption of energy drinks containing metabolic stimulants (e.g., caffeine) in combination with or without alcohol is also accompanied by the increase in OS parameters, including lipid peroxidation (124). The oxidative load caused by alcohol is associated with its biodegradation, which interferes with the mitochondrial and microsomal systems. Ethanol metabolism is directly involved in the production of ROS and RNS. Alcohol consumption reduces the level of GSH and alters antioxidant protection (125). An acute TTS has been described in a 50-year-old man attacked in front of a bar; the level of ethanol in the blood at the time of examination was 400 mg/L (126).

OS is also associated with tobacco smoking and cocaine abuse (127, 128). A number of metabolites (including formaldehyde) formed during the metabolism of cocaine, which caused an acute TTS, ultimately lead to glutathione depletion (128–130). Smoking and alcohol abuse represent risk factors for TTS (7).

TTS attacks have been described in intoxications with herbicides (glyphosate, glufosinate) and insecticides (organophosphates, fungicide) (106). Those are associated with OS, the decrease in glutathione reductase and SOD activity, and the decrease in the concentration of GSH in the plasma and tissues of experimental animals (131). TTS has also been reported in lithium, venlafaxine, risperidone, barbiturates, benzodiazepines, botulinum, or tetanus toxin poisonings (132–136). Other toxins and allergens are mentioned in section Allergic Reactions.

### Photochemical Smog

In a large group of US patients with TTS, the authors identified the late summer months as the period with the highest number of acute attacks (6, 137). This seasonal dependence could be related to the occurrence of so-called photochemical (summer) smog, which was first recognized in Los Angeles during World War II (138). The described form of smog arises in the atmosphere by oxidation of flue gases produced by the combustion of liquid fuels in solar radiation, it promotes the production of nitrogen dioxide and leads to high concentrations of ozone with the participation of other substances in the atmosphere (138, 139). Nowadays, smog episodes are common (not only) in the summer months in various megacities around the world (138). In patients with chronic respiratory disorders, ozone exposure leads to inflammation and lung tissue damage with persistent structural changes (140). About 15% of TTS patients have these comorbidities (13, 102).

## Endogenous Sources of Oxidative Stress in Takotsubo Syndrome

### Physical and Emotional Stress

The term stress was used by Hans Selye to describe the body's inadequate response to mental, emotional, or physical stimuli (141). The reaction known as the adaptation syndrome has three phases (142). The neuroendocrine response to stressors generally involves activation of the autonomic nervous system, activation of the hypothalamic-pituitary-adrenal axis with the cascade of hormonal messengers (e.g., catecholamines, glucocorticoids), and direct nerve stimulation of the adrenal cortex (143–145). This pathway activates the cardiovascular and immune systems, metabolism and potentiates OS associated with a number pathologies of the CNS (24, 143, 144, 146).

#### Physical Stress

Physical stress is described as a trigger in 36–53% of TTS patients (8, 147, 148). They also have higher hospitalization morbidity, mortality and worse long-term prognosis compared to patients with emotional stress (148). Nox, XO, phospholipase A2 (PLA2) and lipoxygenases have been identified as sources of ROS in muscle cells (149). During physical activity, ROS are also produced from non-muscular sources. Endurance and strength requiring physical activity induce the production of pro- and anti-inflammatory cytokines, their inhibitors and chemokines (37, 149). Lipid peroxidation leads to the formation of reactive aldehydes (37, 150). Exercise is generally associated with the oxidation of glutathione in skeletal muscle, blood and liver. However, repeated physical activity enhances glutathione-mediated antioxidant protection, whereas chronic inactivity has the opposite effect (151).

#### Emotional Stress

Emotional stress is mentioned in 21–28% of patients with TTS (8, 147, 148). Various psychological traumas are accompanied by OS in the CNS (144). Compared to patients with acute coronary syndrome, TTS patients have more neurological and psychiatric comorbidities (8). They are also more often neurotic, depressed and anxious (152). Positive emotions such as the so-called “*happy heart syndrome”* have also been described in TTS (118).

Emotional states are the manifestation of the function of the limbic system. The structures of this part of the CNS include the limbic cortex, hippocampal formation including the hippocampus, amygdala, septal area and hypothalamus (145). While the amygdala is associated with the perception of fear and anxiety, with aggression, emotional memory, and social cognition, the cingulate gyrus is involved in the primary autonomic regulation of heart rate and blood pressure (145). The production of neurotransmitters such as dopamine, noradrenaline or serotonin controls the function of the autonomic nervous system and, indirectly, the endocrine, immune and somatic processes (145).

##### Limbic System and Oxidative Stress

For many reasons, the nervous system is highly sensitive to OS (143, 144, 153, 154). In the limbic system, glucocorticoids induce OS by directly accelerating cellular respiration and oxidative phosphorylation (155). Neuronal apoptosis, which was not caused by other steroid hormones, was selectively induced in the dexamethasone-exposed hippocampus (156). Dexamethasone can increase the oxidative state of the hippocampus by up to 200%. The highest increase in ROS production was seen after 4 h of dexamethasone incubation, while the antioxidant activity of GPX was reduced; this effect was mitigated by the antioxidant N-acetyl-L-cysteine (156). Corticosterone has a similar effect (143). In the hippocampus, chronic OS promotes gliogenesis over neurogenesis in neural stem cell progenitors (157).

These facts may be related to the findings of homogeneous structural and functional alterations detected by magnetic resonance imaging in the limbic and central autonomic nervous systems of patients with TTS, who also show increased sympathetic nerve activity associated with decreased spontaneous baroreflex control (158, 159). This may indicate the importance of the brain-heart interaction in TTS (158). The relationship between catecholamines and OS has already been discussed in the section Catecholamines.

### Epileptic Seizures

Secondary TTS was diagnosed in 0.1% of hospitalizations with epilepsy. Caucasians at higher cardiovascular risk were more commonly affected (160). Impaired antioxidant protection, mitochondrial dysfunction, redox-active metals, activation of the arachidonic acid pathway and aging represent critical factors in the genesis of epilepsy. In the CNS, OS is associated with a decrease in GPX activity, a decrease in GPX and GSH/GSSG ratios, and an increase in GSSG, lipid peroxidation, and protein oxidation (161). The high content of polyunsaturated fatty acids in neuronal membranes may be another reason for their high sensitivity to oxidative damage (161, 162). Antiepileptics can also increase OS parameters in erythrocytes, leukocytes, blood, and plasma (161).

### Fenton Reaction

The Fenton reaction can be the source of OS for all TTS conditions associated with bleeding, including injuries, operations, or instrumental procedures. The reaction described by H. J. Fenton takes place in tissues in the presence of hydrogen peroxide, hydroxyl radicals and a suitable ion catalyst (163–165). These are most often iron, copper or cobalt for their ability to cyclically oxidize and reduce in the body (153, 163, 164). Iron is commonly transported and stored in specific proteins that minimize its reactivity. This situation changes under pathological conditions, as blood leaks into tissue outside the vasculature (165). Iron released from erythrocytes catalyzes the autoxidation of physiological compounds or xenobiotics to form highly reactive and toxic substances, which can lead to a number of biological damages (164, 165). TTS has been described after cardiac surgery, shortly after pacemaker implantation, but also after a blood transfusion (165–168). Other case reports related to surgery are also known, some arising between the first and seventh post-operative days (169). Patients with perioperative TTS were younger and less likely to have ECG changes than TTS patients without surgery (169).

### Chronic Diseases

Chronic diseases and comorbidities of TTS such as hypertension, diabetes mellitus, dyslipidemia, metabolic, neurodegenerative and inflammatory diseases, respiratory pathologies and malignancies are associated with OS (13, 24, 32, 33). Some of the drugs used in their treatment are listed in Table 2.

A retrospective analysis of 33,894 patients with TTS and diabetes mellitus (DM) showed the twice lower prevalence of DM compared to the general population (169). This effect is explained by the presence of autonomic neuropathy and the hyposecretion of catecholamines, which accompany more severe forms of DM (169). No potential relationship with the treatment has been assessed yet.

The preferred initial pharmacologic agent for the treatment of type 2 diabetes (first-line therapy) is metformin, which accumulates in the mitochondria at concentrations up to 1,000 times higher than extracellularly (170). Metformin is not metabolized in the body and is excreted unchanged in the urine (Table 2). The drug has been shown to act *via* both AMP-activated protein kinase (AMPK)-dependent and AMPK-independent mechanisms, by the inhibition of mitochondrial respiration, but also perhaps by the inhibition of mitochondrial glycerophosphate dehydrogenase, and by a mechanism involving the lysosome (170, 171). Metformin has a beneficial effect on OS and inflammatory parameters in diabetics (172). Cytochrome inhibitors could have a similar effect (70–72).

### Immune- and Immunopathological Reactions

These reactions accelerate OS through various mechanisms (23, 24, 32, 94, 96). The goal of the inflammatory immune response is to inactivate and eliminate the antigen (173). Acute TTS attacks have been reported in patients with the infections of organ systems, including sepsis, chronic inflammation, autoimmune and demyelinating diseases (174–182). Immunopathological mechanisms have also been detected behind the attacks induced by allopurinol, influenza B, transfusion reaction, or influenza vaccination (56, 57, 183–185).

#### Allergic Reactions

Allergic reactions including anaphylaxis with/without resuscitation, have been described in case reports about TTS (186–190). Various toxins caused acute attacks; some patients have received corticosteroids, tetanus toxoid, or antibiotics during treatment (187, 191–193). A young patient with a shellfish allergy underwent an unspecified dental procedure with the administration of adrenaline before the development of a TTS attack (194). TTS was also described 3 days after contact with the putative antigen which may indicate type IV of immunopathological reaction (delayed type hypersensitivity – DTH) (195).

#### Delayed Type Hypersensitivity

As many as 97% of patients with TTS showed pathological immune reactivity of the DTH type to metals of dental restoration materials (especially amalgam inorganic mercury) evaluated by the lymphocyte transformation test (102). These reactions can be an important hidden (asymptomatic) source of OS. Seventy-nine percentage of patients of this small cohort also had at least one known immunopathological disorder (102). Metals may catalyze the oxidation of catecholamines and accelerate OS (96).

#### Kounis Syndrome

The syndrome described by Kounis and Zavras in 1991 is an “allergic” acute coronary syndrome caused by allergic, hypersensitivity, anaphylactic, or anaphylactoid conditions associated with the activation of platelets, mast cells, macrophages, and T-lymphocytes (196, 197). There are three types of disease: type I is caused by a coronary artery spasm, type II affects patients with the pre-existing atherosclerotic changes in the coronary arteries, where an allergic reaction induces/accelerates the erosion or rupture of the plaque and intracoronary thrombosis. Type III is associated with a hypersensitivity reaction to the coronary stent material leading to “*in stent”* thrombosis (196, 198).

Inflammatory cells activate each other *via* multidirectional signals (196). Inflammatory mediators in these reactions include histamine, platelet-activating factor, arachidonic acid products, neutral proteases, cytokines, and chemokines released during the allergic activation process (196). The triggering factors, similar to TTS, represent food, xenobiotics incl. drugs, poisons, environmental influences, allergic and other pathological conditions (196, 198, 199). In 2009, TTS was described in a patient with asthma and Kounis syndrome (200).

#### Malignancies

According to data from the International Registry, the prevalence of malignancies in patients with TTS is 16.6% (201). The most common are malignancies of the breast, gastrointestinal and respiratory systems, internal genitals and skin. As regards the comorbidities, TTS patients (compared to patients with acute coronary syndrome) were significantly more likely to suffer from COPD, asthma, and neurological pathologies (201). As a trigger of an acute attack, physical stimuli were more frequent than emotional (201). Some children with tumors had TTS too (202, 203). Anticancer drugs as a potential source of OS are listed in Table 2.

## Sarco/Endoplasmic Reticulum—Mitochondria Complex and Calcium Overload—Energy Failure

### Sarco/Endoplasmic Reticulum—Mitochondria Complex

Mitochondria play a central role in the life and death of cells. They provide not only energy metabolism, but also anabolic and catabolic processes, calcium fluxes and various signaling pathways. Mitochondria maintain cellular homeostasis by interacting with ROS and RNS and responding adequately to various stimuli (54). The emission of ROS from mitochondria can induce further release of ROS from neighboring mitochondria, referred to as ROS-induced release of ROS. Mutual communication between different ROS sources represents a (cellular) network of redox signaling (204).

Sarco/endoplasmic reticulum (SER) is a complex organelle formed by a continuous membrane system as a network of interconnected cisterns and tubules (205). Its morphology is determined by integral membrane proteins (e.g., CYP's) and relationships with other organelles such as mytochondria, Golgi apparatus, cell nucleus or cytoskeleton (205, 206). SER consists of 2 parts: the longitudinal SER, composed of tubular structures surrounding the myofilaments, and the junctional SER, which is associated with the transverse tubules to form specialized compartments, where Ca^2+^-induced Ca^2+^ release occurs (204, 207). Close contact points between the SER and mitochondria are controlled by SER-located mitofusin 2 (Mfn2) tethering with Mfn2 and Mfn1 on the outer mitochondrial membrane, respectively, creating microdomains of high Ca^2+^ concentration in the vicinity of mitochondria (204, 207).

Metabolic processes such as glycolysis, the citric acid cycle, β-oxidation of fatty acids, or the metabolism of xenobiotics depend on the continuous recycling of coenzymes (NADH and FADH) into oxidized forms (208). In aerobic organisms, this recovery is ensured by the transport of electrons through the respiratory chain formed by a sequence of redox centers containing CYP's (208). SER complex with mitochondria (the sarco/endoplasmic reticulum - mitochondria complex) regulates protein, lipid, carbohydrate metabolism, steroid synthesis, calcium homeostasis and also myocardial contractility (205, 209).

### Sarco/Endoplasmic Reticulum Stress

Sarco/endoplasmatic reticulum stress occurs when the workload increases (210). This condition is accompanied by the accumulation of degraded proteins in the lumen. It negatively affects cellular homeostasis (206, 211). The cascade of reactions known as the *unfolded protein response* (UPR) serves to restore it (211). With long-term and/or severe SER stress, UPR becomes cytotoxic and can lead to apoptosis (206). Among other things, foci of bound necrosis have been found in myocardial biopsies of TTS patients (113–115).

The main source of OS in the SER are microsomal monooxygenases (so called *MMO system*), the function of CYP 2E1 is emphasized (206). The efficiency/degree of electron transfer binding from NADPH to cytochrome P450 is usually <50–60% and often is as low as 0.5–3.0%. This electron leakage is important for the cellular production of ROS (206). Interactions with various redox mediators such as protein disulfide isomerase, sarco/endoplasmic reticulum oxidoreductin, glutathione, Nox4, NADPH-P450 reductase, and calcium occur during SER oxidative stress (206). These pathways are also associated with pathogenesis of various diseases (206).

### Calcium Overload and Myocardium Energy Failure

OS interferes with the energy and metabolic processes in the cardiomyocyte. Among the numerous mechanisms proposed for myocardial stunning, three appear to be most likely: the generation of ROS, calcium overload, and the excitation-contraction uncoupling caused by an inadequate calcium output from SER were described (51).

Calcium turnover and OS are closely related to the function of mitochondria as the main source of ATP; calcium represents the critical regulator of their function. During β-adrenergic stimulation, cell respiration and Krebs cycle activity are controlled by ADP and mitochondrial Ca^2+^ uptake which regulates the adaptation of cardiomyocytes to continuous acute, chronic, physiological or pathological changes, including increased contractility or apoptosis. Physiological variations of workload lead to mitochondrial Ca^2+^ uptake, which is required to match energy supply to demand, but also to keep the antioxidative capacity in a reduced state to prevent the excessive emission of ROS (204). For the influx of Ca^2+^ into mitochondria is essential the mitochondrial calcium uniporter (MCU), which is the part of a multiprotein complex in the inner mitochondrial membrane (204). Some mitochondria reside in a close apposition to the junctional SER, which is important for excitation–metabolism coupling and apoptosis (207, 212).

Under normal condition, the action potential opens voltage-gated L-type Ca^2+^ channels. The ensuing Ca^2+^ entry triggers even greater release of Ca^2+^ from the SER *via* ryanodine receptors type 2 (RyR2). This Ca^2+^-induced Ca^2+^ release generates a transient increase of the cytoplasmic Ca^2+^ concentration that activates myofilament cross-bridge formation (213, 214). To terminate contraction, Ca^2+^ is removed from the cytoplasm into the SER by the sarco/endoplasmic reticulum calcium ATPase (SERCA) and to the extracellular space by the Na^+^/Ca^2+^ exchanger (NCX) (213–215). While intracellular Ca^2+^ is a mediator of contraction, its influx into the mitochondrial matrix regulates the energy production by modulating pyruvate dehydrogenase and other tricarboxylic acid (TCA) cycle enzymes (216). In the matrix, Ca^2+^ activates Krebs cycle dehydrogenases to regenerate the reduced form of NADH, which donates electrons to the respiratory chain (204). Superoxide generated at complexes I and III of the respiratory chain, is transformed to H_2_O_2_ by SOD and eliminated by GPX and the peroxiredoxin/thioredoxin system (204). Reduced NADPH regenerates glutathione (GSH) from its oxidized form (GSSG) *via* glutathione reductase and thioredoxin reductase, respectively (204).

Under pathological conditions with excitation–contraction uncoupling (e.g., heart failure, TTS), the sarcoplasmic reticulum Ca^2+^ load is reduced (SERCA inhibition) together with the mitochondrial Ca^2+^ concentration (due to the alteration of the MCU and NCX activities); the cytoplasmic Ca^2+^ concentration increases (204, 215, 217). This Ca^2+^ overload activates the mitochondrial permeability transition pore (mPTP), which leads to mitochondrial swelling, outer mitochondrial membrane rupture, and cell death (apoptotic or necrotic) (218). Respiratory chain blockade may occur (219). In the myocardium, the effect is manifested by the depletion of macroergic phosphates with the disruption of myocardial metabolic function and the reduction of contractile force (51, 220).

Changes in intracellular calcium turnover with impaired contractility have been demonstrated in TTS (221). The two homologous proteins sarcolipin (SLN) and phospholamban (PLN) bound to SER are potentially critical regulators of cardiac contractility. While SLN regulates SERCA activity by decreasing calcium affinity, PLN represents a major substrate for PKA in cardiomyocyte. In the unphosphorylated state, PLN is an inhibitor of SERCA, leading to the accumulation of cytoplasmic Ca^2+^ (222, 223). In cardiac biopsies of patients with TTS, SLN expression was significantly increased compared to healthy controls (221). Expression of SERCA2a was significantly down-regulated, PLN dephosphorylation was documented. No changes were demonstrated for NCX and RyR2. This suggests an alteration of Ca^2+^ handling proteins, which might be crucial for the contractile dysfunction (221).

These findings are consistent with the idea of myocardial energy failure in TTS associated with OS (20, 224). Examination of TTS patients with radioisotopes showed an alteration in glucose metabolism during normal myocardial perfusion in an affected left ventricle known as the “*mismatch with inverse flow metabolism”* (225).

## Neurogenic Stunned Myocardium

Neurogenic stunned myocardium (NSM) can be defined as an acute and reversible myocardial dysfunction, occurring after different types of neurologic events, with the participation of the autonomic nervous system (226, 227). That develops during/after subarachnoid hemorrhage (33% of cases), cerebral trauma (22%), ischemic stroke, encephalitis, epilepsy, or tumors (226, 228, 229). Some authors consider NSM as a Takotsubo-like pathology or a secondary TTS (230).

NSM resembles TTS and is explained by limbic-autonomic dysregulation, where the main role is attributed to the insular cortex (231). This visceral-somatic area is one of the least understood parts of the brain. There is a direct structural connection between the insular cortex, the motoric cortex, the limbic system and the autonomic nervous system (232). The severity of NSM depends on the type and severity of the neurologic disease and on involvement of some neurologic structures (insular cortex, hypothalamus, etc.) (230). Diagnostic criteria for NSM are not defined as regards TTS, but include common clinical and echocardiographic characteristics (230). The OS could link the two pathologies.

In patients with TTS, cardiac sympathetic innervation disorder was demonstrated by scintigraphy with I^123^ MIBG in affected areas of the left ventricle (233). This noradrenaline analog was rather poorly absorbed in segments with contractile disorder, unmasking “sympathetic denervation” (233). Similarly, positron emission tomography (PET) with C^11^ hydroxy-ephedrine detected cardiac sympathetic nerve endings. Indicator uptake was reduced in the affected segments, indicating an abnormality of sympathetic innervation as a possible manifestation of stunning in TTS (233).

Cardiac wall contractility disorders in TTS have characteristic patterns corresponding to individual forms of the disease (10). They appear to correlate with the anatomy of sympathetic innervation from the left or right ganglion stellatum or from the caudal ganglion (233–235). In humans, the blockade of the right ganglion stellatum has led to a significant prolongation of the QT interval, which is also found in patients with TTS (3, 236). Recent case reports in a woman with four attacks and three variants of TTS and gastroparesis suggest that stunning could affect a larger neurovisceral area (237).

## Takotsubo Syndrome, Inflammation, Histological Findings

### Takotsubo Syndrome and Inflammation

Cardiac magnetic resonance (CMR) imaging in the acute phase of TTS showed the typical pattern of left ventricle dysfunction, corresponding to wall motion abnormalities, markers for myocardial inflammation with hyperemia and tissue edema, and the absence of significant necrosis/fibrosis (238–240). During follow-up, there is a complete normalization of the left ventricular ejection fraction and inflammatory parameters (238). According to the findings from positron emission tomography/computer tomography (PET CT), the coronary flow reserve and myocardial blood flow in TTS are disrupted globally and not only in the areas of the contractile disorder (241). Magnetic resonance with multiparametric myocardial imaging detected inflammatory macrophage infiltration in the affected areas (242). In the blood of these patients, classical CD14++ CD16- monocytes and serum concentrations of interleukin-6 and chemokine (C-X-C motif) ligand 1 were increased simultaneously whereas intermediate CD14++CD16+ and non-classic CD14+CD16++ monocytes were reduced (242).

Some of these changes persisted for at least 5 months, suggesting a low intensity of chronic inflammation in patients with TTS (242). Monocyte activation is also associated with OS – see sections Oxidative Stress in Medicine and Catecholamines and Oxidative Stress, respectively. In another study, patients in the acute phase of TTS had higher concentrations of interleukins 2, 4, 10, TNF-α, IFN-γ, and EGF, but lower levels of interleukin 6 compared to individuals with acute coronary syndrome (243).

### Histological Findings in Takotsubo Syndrome

Histological findings in patients with TTS were described from endomyocardial biopsies, but also from autopsies. In biopsies taken from the myocardium in the acute phase of TTS, sets of genes controlling the antioxidant response were triggered by OS by the induction of SOD, CAT and GPX1 (17). Areas of interstitial edema with irregular infiltrates of monocytes, macrophages, rarely with polymorphonuclear lymphocytes, mast cells and eosinophils are present in the tissue sections (114, 115). The activation of immune cells is associated with OS (94–96). Tissue edema could be the final manifestation of energy production blockade (see section Calcium Overload and Myocardium Energy Failure). Myocytes or groups of myocytes show increased eosinophilic staining, ruptures, and necrosis (114). Epicardial impairment, inflammatory changes, and contraction bands distinguish TTS from coagulation necrosis in acute myocardial infarction (114, 115). Both ventricles may be affected, there is no myocardial fibrosis or correlation with coronary anatomy (114).

In six of the nine fatal cases of TTS, a disease with an immunopathological background was present as (chronic) comorbidity - rheumatoid arthritis, Hodgkin's disease, multiple myeloma, bronchial asthma, and pneumonia (114). Acute inflammation, sepsis or adrenaline intake were the most common triggers of TTS (114). These pathologies are also associated with OS (23, 24, 33).

## Takotsubo Syndrome and Antioxidants

Plasma concentrations of certain biomarkers well reflect the redox state in tissues including the heart (244). In women of a small TTS group, high prevalence of vitamin D insufficiency was found associated with worse hemodynamic parameters (245). In addition to interfering with calcium metabolism, vitamin D is also an important antioxidant.

The administration of α-lipoic acid vs. placebo in TTS patients resulted in the significant reduction in CRP, TNF-α and nitrotyrosine (OS marker) (246). This effect is explained by the adjustment of sympathetic-vagal vegetative alteration or the regulation of sympathetic tone (246, 247). Alpha-lipoic acid as an antioxidant plays a crucial role in mitochondrial dehydrogenase reactions, reacts with various RS, protects membranes by interacting with vitamin C and glutathione, which in turn recycles vitamin E (248). Its administration has had beneficial effects in many OS models (248). Another antioxidant, N-acetyl-L-cysteine, reduced OS levels in the hypocampus upon exposure to dexamethasone (156). Further experimental works has shown a beneficial effect of other antioxidants (SOD, ascorbic acid, α-tocopherol) on the synthesis of adrenochrome (100, 101).

Interactions with glutathione and substances/enzymes related to its metabolism have been repeatedly reported in the above-mentioned sources of OS, including physical and mental stress. Glutathione as a product of the methionine-homocysteine cycle is a major endogenous antioxidant (122). The cytoplasm, mitochondria and cell nuclei contain mainly a reduced form of glutathione (GSH) representing up to 99% of total glutathione pool. SER with MMO system contains up to 30% of glutathione in oxidized form as GSSG. A reduced GSH/GSSG ratio is considered an indicator of OS (122). Reduced glutathione performs antioxidant, detoxifying and chelating functions (122). The methionine-homocysteine cycle also provides methyl group transfer for COMT-catalyzed reactions involved in the biodegradation of catecholamines (122, 249). The reduced efficiency of this system could lead to alterations in the biotransformation of catecholamines, including oxidized forms.

Significantly low concentrations of total glutathione were found in the blood of healthy individuals of various ages, especially in the age group of 60–79, which is most often affected by TTS (7, 8, 36). These individuals may be at the risk due to reduced ability to maintain glutathione-mediated metabolic and detoxification pathways (36, 122). Patients with chronic diseases also have significantly lower GSH levels (250). Under OS stress, lower glutathione concentrations are easier to deplete. In contrast, in women of childbearing age, a positive correlation was found between blood estrogen and GSH levels, and CAT activity, respectively (251).

## Discussion

**Figure 1:**
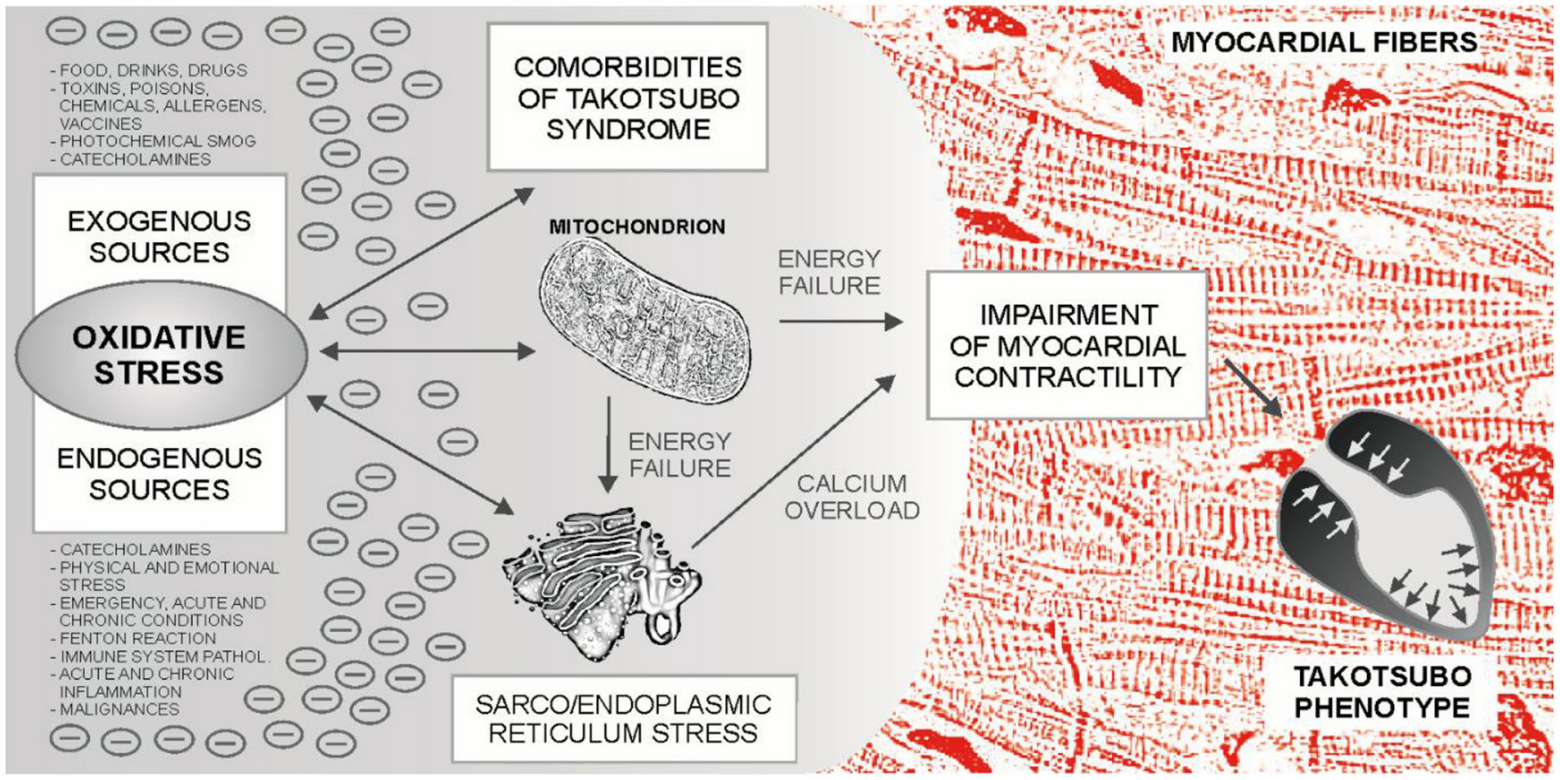
Sources and effects of oxidative stress (OS) in Takotsubo syndrome (TTS). Prior to the development of an acute attack of TTS, various sources of OS can be identified, which can be combined with each other. The main sources of oxidative stress in the body are mitochondria and the mitochondria - sarco/endoplasmic reticulum complex, which are involved in xenobiotic metabolism, calcium turnover and myocardial contractility, among others. Catecholamines can act as metabolic accelerators in this system. Finally, accumulated oxidative stress could lead to the blockade of energy production, disruption of calcium turnover in the cardiomyocyte, impaired contractility, and to the development of the Takotsubo phenotype.

**Figure 2:**
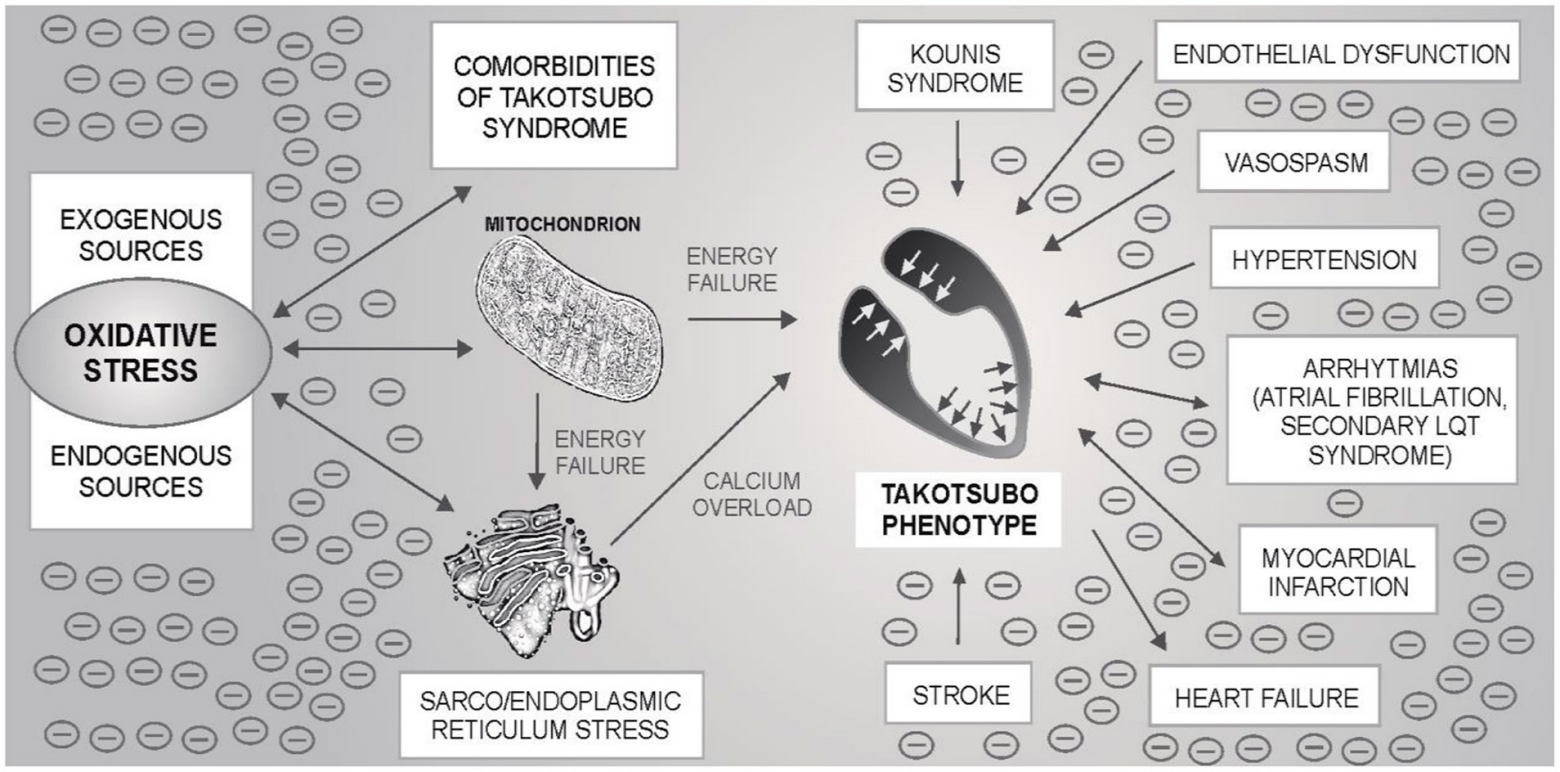
Possible relationship between oxidative stress (OS), mitochondria-sarco/endoplasmic reticulum complex, Takotsubo syndrome (TTS) and other cardiovascular pathologies. By the mechanisms described in the text, OS can lead to the blockade of energy production and calcium overload of myocytes with the subsequent development of an acute attack of TTS. OS is also associated with some TTS comorbidities - one of the most common is hypertension, but also e.g., atrial fibrillation. Secondary long QT syndrome and/or heart failure may be present in TTS. Stroke (ischemic or hemorrhagic) and, exceptionally, myocardial infarction have been described as the triggers of an acute TTS attack. If TTS is associated with OS, an acute attack itself can be an important source of OS. The oxidation load is likely to be potentiated. OS may affect other cardiac structures, incl. coronary (micro) circulation or cardiac conduction system. Endothelial dysfunction and vasospasm have previously been mentioned as possible pathophysiological mechanisms in TTS. Under these circumstances, an acute TTS attack could trigger an acute myocardial infarction. Kounis syndrome is an “allergic” acute coronary syndrome caused by allergic, hypersensitivity, anaphylactic or anaphylactoid conditions. There are three types of the disease: caused by a coronary artery spasm, by the acceleration of the pre-existing atherosclerotic changes in the coronary arteries, and by hypersensitivity reactions to the coronary stent material leading to “*in stent”* thrombosis, respectively.

Takotsubo syndrome is one of the greatest mysteries of cardiology in the last 30 years. Based on the evidence presented, it can be hypothesized that TTS may occur in neuro-myocardial areas with escalated OS, typically in post-menopausal women. For the development of an acute attack of TTS, the OS could represent an essential metabolic regulator as a “*conditio sine qua non”* ultimately leading to the sarco/endoplasmic reticulum – mitochondria complex stress with blockade of energy production, calcium overload and Takotsubo phenotype – Figure 1. Subsequent adjustment of metabolic functions may lead to restoration of dynamic equilibrium. An example could be the recent case report describing the development of three forms of TTS in a single patient with intracerebral hemorrhage, where several sources of OS can be identified (252). Possible relationships between TTS, OS and cardiovascular pathologies are shown in Figure 2.

Our approach can help in the complex description of the circumstances leading to an acute attack. OS could link three similar heart pathologies with similar or identical triggers – Takotsubo syndrome, Kounis syndrome and neurogenic stunned myocardium.

The correctness of this hypothesis should be confirmed by a detailed analysis of clinical cases, direct biochemical evidence in patients with acute TTS attack and subsequent modeling of TTS in the experiment. TTS can be a much more complex pathology than it currently seems to be.

## Data Availability Statement

The original contributions presented in the study are included in the article/supplementary material, further inquiries can be directed to the corresponding author.

## Author Contributions

All authors listed have made a substantial, direct and intellectual contribution to the work, and approved it for publication.

## Funding

This work was supported by the Ministry of Health of the Czech Republic – conceptual development of research organization (FNBr, 65269705; funding was given to University Hospital Brno).

## Conflict of Interest

The authors declare that the research was conducted in the absence of any commercial or financial relationships that could be construed as a potential conflict of interest.

## Publisher's Note

All claims expressed in this article are solely those of the authors and do not necessarily represent those of their affiliated organizations, or those of the publisher, the editors and the reviewers. Any product that may be evaluated in this article, or claim that may be made by its manufacturer, is not guaranteed or endorsed by the publisher.

## Abbreviations

AMP: adenosine monophosphate
AMPK: AMP-activated protein kinase
ADP: adenosine diphosphate
ATP: adenosine triphosphate
βAR-GRK2: βadrenergic receptor Gprotein-coupled receptor kinase 2 complex
βAR: βadrenergic receptors
BOS: basal oxidative stress
cADPR: cyclic adenosine monophosphate ribose
CAT: catalase
CMR: cardiac magnetic resonance
CNS: central nervous system
COMT: catechol-o-methyltransferase
COPD: chronic obstructive pulmonary disease
CRP: C reactive protein
CYP: cytochrome P450
DM: diabetes mellitus
DNA: deoxyribonucleic acid
DOS: dietary oxidative stress
DTH: delayed-type hypersensitivity
EGF: epidermal growth factor
ETC: electron transport chain
FADH: flavin adenine dinucleotide hydrogen
FFA: free farry acids
FR: free radicals
GPX: glutathione peroxidase
GSH: reduced glutathione
GSSG: glutathione disulfide
GST: glutathione S-transferase
HER2: human epidermal growth factor receptor 2
HIV: human immunodeficiency virus
HOS: high intensity oxidative stress
IFN-γ: interferon gamma
IOS: intermediate intensity oxidative stress
LOS: low intensity oxidative stress
MAO: monoamino oxidase
MCU: mitochondrial calcium uniporter
MIBG: meta-iodobenzylguanidine
Mfn1, 2, SER-located mitofusin 1: 2
MMO system: microsomal monooxigenase system
mPTP: mitochondrial permeability transition pore
NADH: nicotinamide adenine dinucleotide hydrogen
NADPH: nicotinamide adenine dinucleotide phosphate hydrogen
NCX: Na^+^/Ca^2+^ exchanger
NOS: nutritional oxidative stress
Nox: NADPH oxidase
NSAID: non-steroid antiinflammatory drug
NSM: neurogenic stunned myocardium
Nrf2: Nuclear factor erythroid-2-related factor 2
OS: oxidative stress
PET CT: positron emission tomography/computer tomography
PKA: protein kinase A
PLA2: phospholipase A2
PLN: phospholamban
POS: post-prandial oxidative stress
ROS: reactive oxygen species
RNS: reactive nitrogen species
RS: reactive species
RyR2: ryanodine receptors type 2
SER: sarco/endoplasmic reticulum
SERCA: sarco/endoplasmic reticulum calcium ATPase
SLN: sarcolipin
SOD: superoxide dismutase
SOS: severe (strong) oxidative stress
SNRI: serotonin-norepinephrin reuptake inhibitor
TNF-α: tumor necrosis factor alpha
TTS: Takotsubo syndrome
UPR: unfolded protein response
XO: xanthine oxidase.
